# The Role of Index of Microcirculatory Resistance in Left Anterior Descending Artery ST Segment Elevation Myocardial Infarction Patients after Primary Percutaneous Coronary Intervention

**DOI:** 10.3390/jcm13071989

**Published:** 2024-03-29

**Authors:** Seong Huan Choi, Sung Gyun Ahn, Myeong Ho Yoon, Kyoung-Woo Seo, Ki-Jeung Lee, Sung Woo Kwon, Sang-Don Park, Seong-Ill Woo

**Affiliations:** 1 Division of Cardiology, Department of Medicine, Inha University Hospital, Incheon 22332, Republic of Korea; seonghuan2@hanmail.net (S.H.C.);; 2 Division of Cardiology, Department of Internal Medicine, Yonsei University Wonju College of Medicine, Wonju 26426, Republic of Korea; 3 Department of Cardiology, Ajou University School of Medicine, Suwon 16499, Republic of Korea

**Keywords:** STEMI, IMR, all-cause mortality

## Abstract

**Background**: Our aim was to assess the relationship of the index of microvascular resistance (IMR) in left anterior descending (LAD) artery involved STEMI patients. **Methods**: Data of 316 STEMI patients who had undergone primary percutaneous coronary intervention (PCI) were collected from three cardiovascular centers from 2005 to 2015. In total, 246 patients with LAD STEMI were enrolled for IMR evaluation. Patients were divided into two groups respective of the cut-off IMR value of 30. All-cause mortality, left ventricular function, improvement of systolic function, and cardiac biomarkers were analyzed and compared. **Results**: A total of 246 patients were enrolled. The number of patients in the IMR above 30 group was 93 and below 30 was 153. The mean ages for each group were 57.91 ± 11.99 and 54 ± 10.63, respectively. The peak creatinine kinase (CK) (3936.85 ± 2827.32 IU/L vs. 2218.08 ± 2310.41 IU/L, *p* < 0.001) and CKmb (336.15 ± 195.08 mg/mL vs. 231.53 ± 179.53 mg/mL, *p* < 0.001) levels were higher for an IMR above the 30 group. The left ventricular ejection fraction (LVEF) (44.57 ± 6.685% vs. 47.35 ± 8.17%, *p* = 0.006) and improvement of LVEF (2.81 ± 7.135% vs. 5.88 ± 7.65%, *p* = 0.004) was lower in the IMR above 30 group. All-cause mortality (7.5% vs. 1.3%, *p* = 0.012) was higher in the IMR above 30 group, and a Cox regression analysis showed that an IMR above 30 was a poor prognostic factor regarding all-cause mortality (HR: 5.151, 95% CI 1.062–24.987, *p* = 0.042) even after adjusting for classical clinical risk factors. **Conclusions**: An elevated IMR value represented larger infarct size, more severe LV dysfunction, and higher mortality in LAD STEMI patients after successful PCI.

## 1. Introduction

The technological advancement of drug eluting stents [1] and increasing emphasis on the importance of timely percutaneous coronary intervention has given ST segment elevation myocardial infarction (STEMI) patients higher survival rates and enhanced their quality of life [2,3]. Through decades of research, tangible risk factors and prognosticators associated with poor clinical outcomes were identified [4,5,6]. The rapidly growing field of bio-medical technology allowed researchers access to highly sophisticated physiological examination tools which have further broaden the spectrum of clinical research. The index of microcirculatory resistance (IMR) was first introduced via an animal study nearly two decades ago [7]. With relative ease of reproducibility, this quickly extended to human trials and demonstrated that an elevated IMR was associated with higher mortality in stable-angina patients [8]. A recent study showed that the IMR value obtained in STEMI patients correlated well with their infarction sizes and microvascular obstruction [9]. However, despite the abundance of studies focusing on the role of the IMR value in a variety of cardiovascular diseases, investigative study regarding a certain specified epicardial vessel is sparse. Indeed, functional flow reserve (FFR), which is another physiological apparatus utilizing a pressure wire technique to assess the ischemic burden on coronary arteries, has shown that despite the validated role of global FFR, discrepancies regarding lesion length, type, and location do exist amongst the study population. Furthermore, these studies have shown that the least ‘visual–functional’ mismatch was observed in the left anterior descending (LAD) stenosis [10,11]. Therefore, we sought to look into the clinical implications of IMR values in a LAD STEMI patient who underwent primary PCI.

## 2. Materials and Methods

### 2.1. Study Design and Patient Selection

**Figure 1:**
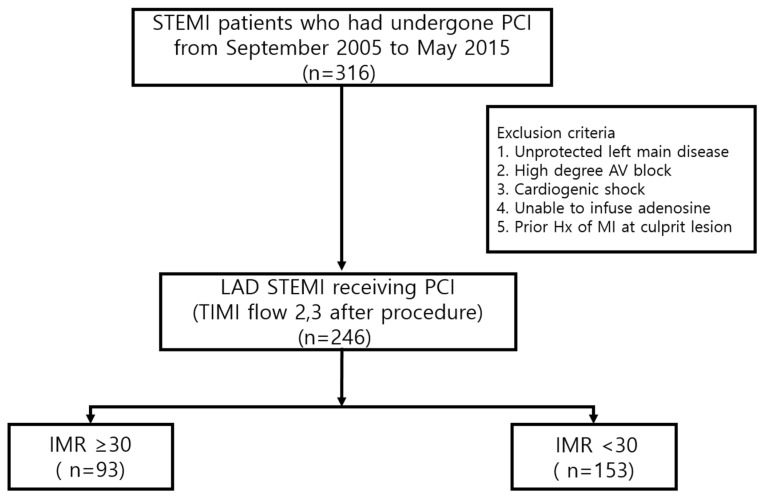
Study protocol and flow chart. STEMI: ST segment elevation myocardial infarction; PCI: percutaneous coronary intervention; LAD: left anterior descending; AV: atrioventricular; MI: myocardial infarction; TIMI: thrombolysis in myocardial infarction; IMR: index of microvascular resistance.

Our current study was a retrospective multi-center study comprising three tertiary hospitals from Korea. Consecutive STEMI patients who had undergone primary PCI from September of 2005 to May of 2015 were enrolled. The inclusion criteria were patients with (1) LAD involved STEMI; (2) TIMI grade 2.3 after PCI. The exclusion criteria were (1) unprotected left main disease; (2) high degree atrioventricular (AV) block; (3) cardiogenic shock; (4) condition not availing adenosine infusion; (5) and prior history of myocardial infarction (MI) at designated culprit vessel (Figure 1).

### 2.2. Definition of Variables and Measurements

STEMI was defined when a patient presents with typical chest pain with electrocardiograms (ECG) showing (1) newly developed ST segment elevation of two or more continuous leads with ST segment greater than 0.1 mV elevation in all leads other than V2 or V3; (2) in leads V2 or V3, ST segments greater than 0.2 mV for men and 0.15 mV for women [12]. Patients with systolic blood pressure higher than 140 mmHg, diastolic blood pressure over 90 mmHg, or prior use of antihypertensive medication were defined as having hypertension. Diabetes mellitus (DM) was defined according to the following criteria (1) prescription of hypoglycemic agents or insulin; (2) fasting glucose level above 126 mg/dL or glycosylated hemoglobin (HbA1c) above 6.5%; and (3) untreated hyperglycemia. The definition of dyslipidemia was as follows (1) total cholesterol above 240 mg/dL; (2) low-density lipoprotein (LDL) cholesterol above 130 mg/dL; (3) high-density lipoprotein (HDL) cholesterol below 40 mg/dL; (4) triglycerides level above 200 mg/dL; and previous prescription of lipid-lowering agents. A patient who was actively smoking or had smoked up until 1 month prior to primary PCI was considered as a smoker. The TMP grade was measured at each institution, respectively. Three interventional cardiologists evaluated the coronary angiogram, and grading was established only when the three experts came to a coherent conclusion. An IMR cut-off value of 30 was established utilizing a previous study’s protocol [13,14,15,16]. The patients were divided into groups with an IMR of above or below 30 U. 

### 2.3. Intra Coronary Physiologic Measurements

Intra coronary physiologic values were measured at the culprit vessel after successful PCI using a pressure/temperature sensor-tipped wire (Radi Medical system, Uppsala, Sweden). The wire was calibrated before entering the patient’s arterial system and equalized at the tip of the guiding catheter and advanced two thirds distally to the culprit vessel. In total, 3 mL of room-temperature normal saline was infused to measure the baseline mean transit time. After 140 μg/kg/min intravenous adenosine infusion for maximal hyperemia, another three saline injections were administered to measure the hyperemic transit time. The IMR was defined by multiplying the distal coronary pressure at maximal hyperemia with the hyperemic mean transit time. The coronary flow reserve (CFR) was obtained by dividing the baseline mean transit time by the hyperemic mean transit time. The fractional flow reserved (FFR) was the ratio between the mean aortic pressure and the distal coronary pressure during hyperemia. 

### 2.4. Endpoints Determination and Follow-Up Data Acquisition

The primary end point was all-cause mortality regarding their IMR value. Clinical parameters which can mirror the infarct size and disease severity such as cardiac biomarkers and left ventricular ejection fraction (LVEF), along with conventional risk factors for cardiovascular disease (age, body mass index, hypertension, diabetes mellitus), were also analyzed. Follow up data were acquired through routine telephone interviews and electronic medical record review. 

### 2.5. Statistical Analysis

Continuous data were presented as means ± standard deviations and categorical data as percentages or absolute numbers. Continuous data were analyzed using analysis of variance and categorical data using the chi-square test to assess the differences between the two groups. A Cox proportional hazard regression analysis utilizing the backward elimination technique was performed to analyze the association between IMR and clinical outcome. Hazard ratios (HRs) were calculated as an estimate of the risk associated with a particular variable with 95% confidence intervals (CIs). The proportional hazard assumptions of the HR in the Cox proportional hazard models were graphically inspected in the “log minus log” plot and tested using Schoenfeld residuals. The omitted columns represented multivariate parameters that were not statistically significant. The Kaplan–Meier (KM) method was used to estimate event-free survival. All analyses were performed using SPSS (version 19.0; SPSS, Chicago, IL, USA) and SAS (version 9.3; SAS Institute, Cary, NC, USA). The statistical significance level was set at *p* < 0.05.

## 3. Results

### 3.1. Baseline Characteristics

The mean follow up period was 1974.52 ± 1092.248 days. The mean age for the total population was 55.47 ± 11.30. Male gender along with current smoking status (78.5%) was predominant amongst the total population (87.8%). Peak creatinine kinase (CK), CKmb, and Trop-I levels were (2808.36 ± 2623.66), (270.66 ± 191.93), (66.13 ± 73.53), respectively. The mean left ventricular ejection fraction (LVEF) was 46.31 ± 7.75. The mean ages for IMR above and below 30 were 57.91 ± 11.99 and 54 ± 10.63, *p* = 0.008, respectively. Clinical and classical risk factors such as gender, body mass index (BMI), systolic and diastolic blood pressure, heart rate, hypertension (HTN), and diabetes mellitus (DM) showed no difference between the two groups. The prevalence of dyslipidemia was higher for the IMR below 30 group (30.1% vs. 56.5%, *p* < 0.001). The peak CK and CKmb levels were significantly higher for the IMR above 30 group (3936.85 ± 2827.32 IU/L vs. 2218.08 ± 2310.41 IU/L, *p* < 0.001) (336.15 ± 195.08 mg/mL vs. 231.53 ± 179.53 mg/mL, *p* < 0.001). The baseline LVEF and the improvement of LVEF after PCI was both lower and smaller in the IMR above 30 group (44.57 ± 6.685% vs. 47.35 ± 8.17%, *p =* 0.006) (2.81 ± 7.135% vs. 5.88 ± 7.65%, *p* = 0.004). The medication profile including anti platelet agents, angiotensin receptor blocker (ARB)/angiotensin converting enzyme inhibitor (ACEi), b-blocker, and statin did not show any statistical differences between the two groups (Table 1). 

### 3.2. Angiographic Characteristics

Single vessel disease was most prominent for both the IMR above and below 30 groups (57.6% and 71.4%, *p* = 0.017). The proportion of TIMI flow 0 before PCI was significantly higher for the IMR above 30 group (57.3% vs. 36.7%) and vice versa for the proportion of TIMI flow 3 (2.2% vs. 15.1%). The drug eluting stent (DES) diameter and length did not show any statistically significant differences. TIMI myocardial perfusion (TMP) grade 3 was significantly lower in the IMR above 30 group (36.8% vs. 63.6%) (Table 2). 

### 3.3. Primary Endpoint and LVEF

**Figure 2:**
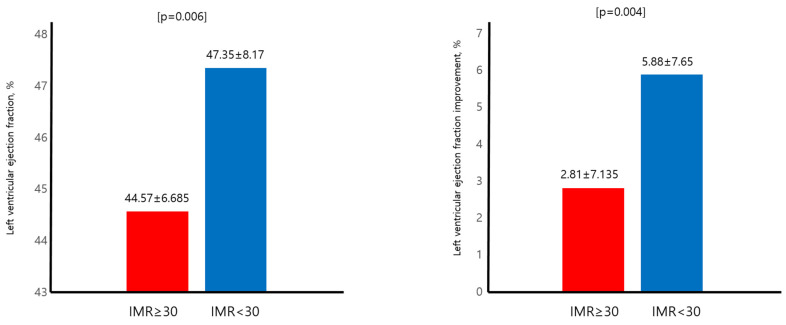
Baseline LV function and improvement of LV function. LV: left ventricular; IMR: index of microvascular resistance.

**Figure 3:**
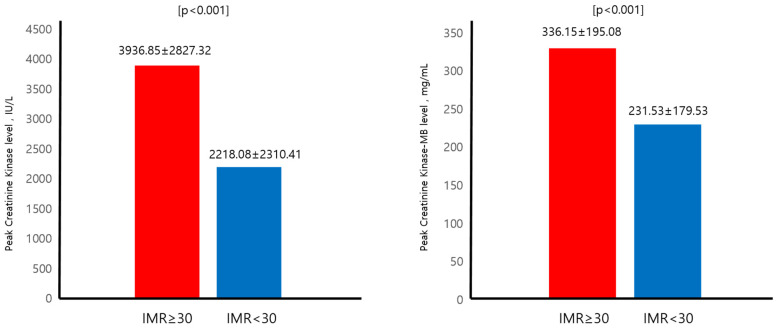
Peak CK and CKmb level with respect to IMR. CK: creatinine kinase; IMR: index of microvascular resistance.

**Figure 4:**
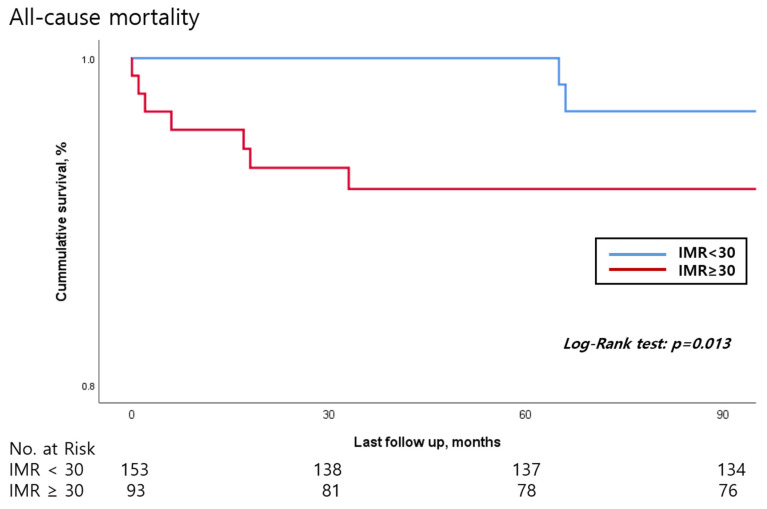
Kaplan meier survival curve regarding all-cause mortality. IMR: index of microvascular resistance.

Patients with an IMR above 30 had higher incidences of all-cause mortality (7.5% vs. 1.3%, *p* = 0.012). The baseline LVEF was significantly lower in the IMR above 30 patients (44.57 ± 6.685% vs. 47.35 ± 8.17%, *p* = 0.006). Improvement of LVEF after successful PCI also differed between the two groups and the IMR above 30 group showed a smaller LV systolic function improvement compared to the IMR below 30 patients (2.81 ± 7.135% vs. 5.88 ± 7.65%, *p* = 0.004). In Figure 2, the peak CK and CKmb level were also higher in the IMR above 30 patients (3936.85 ± 2827.32 IU/L vs. 2218.08 ± 2310.41 IU/L, *p* < 0.001) (336.15 ± 195.08 mg/mL vs. 231.53 ± 179.53 mg/mL, *p* < 0.001) In Figure 3, univariate and multivariate Cox regression models showed that even after adjusting for clinical risk factors associated with mortality, IMR above 30 (HR 5.038, 95% CI 1.039–24.442, *p* = 0.045) was a poor prognostic factor regarding all-cause mortality. In Table 3, the KM curve demonstrated that the IMR above 30 patients had lower event-free survival compared to the IMR below 30 patients with respect to all-cause mortality (Figure 4). 

## 4. Discussion

Our current study evaluated the prognostic effect of the IMR value in completely re-vascularized LAD STEMI patients. Groups were divided into IMR above 30 and IMR below 30. Baseline characteristics showed that the IMR above 30 patients had higher peak cardiac enzyme levels, a lower baseline LVEF, and smaller improvement of LVEF after index PCI which translates to worse LV dysfunction along with higher mortality. 

Ever since the introduction of coronary intervention [17], the prognosis of coronary artery disease (CAD) patients has shifted in trajectory [18]. The evolution of DES accompanied by the development of coronary intervention devices have allowed room for improved outcomes not only for the short-term prognosis but also long-term survival. Before these technological advancements, many researchers have delved into the idea of risk stratification regarding mortality in STEMI patients, and thanks to the endeavors from many past researchers [4,5,6], comprehensive guidelines to ensure greater outcomes were established [19,20]. However, despite the implementation of these aforementioned scoring systems to further enhance patient survival, the mortality rate of STEMI patients is still relatively high. Therefore, physiologic parameters measured during catheterization of the coronary artery drew attention from interventional cardiologist [21]. The concept of CFR was first introduced to distinguish high-risk CAD patients from regular patients [22]. However, although CFR was able to identify high-risk CAD patients, a considerable amount of inconsistency was observed regarding the extend of CAD and CFR’s value. Furthermore, despite CFR’s predictive value, it did not represent the microcirculatory functional capacity. The IMR, which was able to measure segmental arterial microcirculatory resistance, showed prognostic influence over a wide range of the coronary disease spectrum [9,23,24]. Recently, many studies have been published emphasizing the clinical implications and possible role of IMR as a risk-assessing tool regarding prognosis in STEMI patients, and our current study was able to reappraise the current notion [25,26,27]. Most recent studies evaluated the influence of IMR on the entire spectrum of acute coronary syndrome (ACS) with diverse culprit lesions. However, data on selective sub analysis of a single designated pericardial coronary artery (in our case the LAD) is relatively sparse. In our study, STEMI patients with LAD culprit lesion were enrolled for analysis and an IMR value above 30 was associated with a poor prognosis regarding all-cause mortality along with more severe LV systolic dysfunction and smaller capacity for LV function recovery. Therefore, for STEMI patients who had received successful PCI and were hemodynamically eligible for IMR evaluation, IMR can provide useful clinical information since it is related to LV systolic function and ultimately long-term mortality. 

Our study has some limitations. Firstly, the sample size for the study population is too small. Although three tertiary hospitals were involved in patient enrollment, only 316 patients were initially included. This study was a sub-analysis of LAD STEMI; therefore, the total study population was even smaller. Furthermore, due the nature of our study design being retrospective the Kaplan–Meyer estimation of all-cause mortality might be over represented. Secondly, the mortality rate was very low due to the fact that all patients had received successful PCI at LAD STEMI with relatively acceptable TIMI flow which was a prerequisite for IMR analysis. Thirdly, this was a retrospective study comprising only the Korean population; therefore, a multi-national study with a larger study population is warranted.

## 5. Conclusions

An IMR above 30 was associated with worse all-cause mortality in LAD STEMI patients who had received successful PCI. An IMR above 30 was also associated with larger infarct size, lower LV systolic function, and smaller LV function recovery. 

## Figures and Tables

**Table 1:** Baseline characteristics.

Baseline Characteristics	Total Population (246)	IMR ≥ 30 (93)	IMR < 30 (153)	*p* Value
Age, years	55.47 ± 11.30	57.91 ± 11.99	54 ± 10.63	0.008
Gender (male), n%	216 (87.8%)	76 (81.7%)	140 (91.5%)	0.023
BMI, kg/m^2^	24.45 ± 3.01	24.52 ± 3.50	24.39 ± 2.68	0.741
SBP, mmHg	133.44 ± 23.12	136.93 ± 25.56	131.55 ± 21.56	0.117
DBP, mmHg	82.64 ± 15.95	84 ± 18.1	81.91 ± 14.74	0.377
Heart rate, bpm	79.06 ± 15.15	77.61 ± 13.77	79.83 ± 15.83	0.325
HTN	91 (36.8%)	37 (39.8%)	54 (35.1%)	0.456
DM	61 (24.7%)	24 (25.8%)	37 (24%)	0.753
Dyslipidemia	115 (46.6%)	28 (30.1%)	87 (56.5%)	<0.001
Smoking	194 (78.5%)	70 (75.3%)	124 (80.5%)	0.33
Prior PCI	2 (1.3%)	1 (1.5%)	1 (1.1%)	0.831
Door-to-balloon time, min	81.27 ± 89.29	80.45 ± 82.55	81.78 ± 93.49	0.911
Symptom-to-balloon time, min	357.47 ± 917.75	412.05 ± 1040.84	323.54 ± 833.71	0.469
Symptom-to-door time, min	322.10 ± 1140.32	386.72 ± 1248.12	275.10 ± 1059.958	0.553
Serum creatinin	1.00 ± 0.29	0.98 ± 0.34	1.02 ± 0.26	0.418
Ntpro BNP	929.51 ± 3719.96	1501.97 ± 5059.89	547.87 ± 2455.89	0.28
Peak CK, IU/L	2808.36 ± 2623.66	3936.85 ± 2827.32	2218.08 ± 2310.41	<0.001
Peak CK-MB, mg/mL	270.66 ± 191.93	336.15 ± 195.08	231.53 ± 179.53	<0.001
Peak Trop-I, ng/mL	66.13 ± 73.53	76.15 ± 82.15	60.39 ± 67.74	0.122
IMR	29.70 ± 20.68	49.92 ± 20.91	17.56 ± 5.44	<0.001
All-cause mortality	9 (3.7%)	7 (7.5%)	2 (1.3%)	0.012
Medication				
clopidogrel	210 (85.3%)	80 (86%)	130 (84.4%)	0.732
ticagrelor	30 (12.2%)	10 (10.7%)	20 (13%)	0.393
prasugrel	5 (2%)	1 (1%)	4 (3%)	0.299
ARB/ACEi	127 (51.2%)	46 (49.5%)	81 (52%)	0.842
B-blocker	132 (53.2%)	46 (49.5%)	86 (56%)	0.231
Statin	138 (55.65)	50 (53.7%)	88 (57%)	0.452

Data are expressed as number (%) or mean ± standard deviation. BMI = body mass index; SBP = systolic blood pressure; DBP = diastolic blood pressure; HTN = hypertension; DM = diabetes mellitus; PCI = percutaneous coronary intervention; NTpro BNP = B-type natriuretic peptide; CK = creatine kinase; CK-MB = creatine kinase myocardial band; IMR = index of microcirculatory resistance; ARB: angiotensin receptor blocker; ACEi: angiotensin converting enzyme inhibitor.

**Table 2:** Angiographic characteristics.

Baseline Characteristics	IMR ≥ 30 (93)	IMR < 30 (153)	*p* Value
Number of vessels, n (%)			0.017
1	53 (57.6%)	110 (71.4%)	
2	35 (38%)	33 (21.4%)	
3	4 (4.3%)	11 (7.1%)	
TIMI grade before PCI, n (%)			0.001
0	51 (57.3%)	51 (36.7%)	
1	19 (21.3%)	29 (20.9%)	
2	17 (19.1%)	38 (27.3%)	
3	2 (2.2%)	21 (15.1%)	
DES characteristics			
Stent diameter, mm	3.19 ± 0.38	3.18 ± 0.32	0.81
Stent length, mm	26.61 ± 9.06	25.37 ± 9.9	0.327
TMP grade after PCI, n (%)			<0.001
0	8 (9.2%)	0 (0%)	
1	16 (18.4%)	1 (0.8%)	
2	31 (35.6%)	47 (35.6%)	
3	32 (36.8%)	84 (63.6%)	
TIMI grade after PCI, n (%)			<0.001
0/1	0	0	
2	22 (25.6%)	4 (3.1%)	
3	64 (74.4%)	123 (96.9%)	

TIMI: thrombolysis in myocardial infarction; DES: drug eluting stent; TMP: TIMI myocardial perfusion; PCI: percutaneous coronary intervention.

**Table 3:** Univariate and multivariate Cox regression model for all-cause mortality.

Variables	Univariate Analysis			Mutivariate Analysis		
	HR	95% CI	*p* Value	HR	95% CI	*p* Value
Age	1.056	0.999–1.117	0.055			
Gender	0.488	0.101–2.350	0.371			
Dyslipidemia	1.047	0.280–3.909	0.946			
BMI	0.829	0.684–1.005	0.057			
Hypertension	3.719	0.929–14.879	0.063			
Diabetes	2.499	0.670–9.319	0.173			
Current smoking	0.492	0.123–1.973	0.317			
Serum CK mb	1.001	0.998–1.004	0.413			
Serum trop	1.008	1.001–1.015	0.028			
Multi vessel disease	1.667	0.447–6.212	0.447			
Initail TIMI flow 0	2.531	0.633–10.122	0.189			
Symptom to balloon time	1.000	0.994–1.002	0.789			
Door to balloon time	0.987	0.960–1.016	0.386			
LV ejection fraction	0.871	0.784–0.968	0.01	0.856	0.749–0.980	0.024
IMR ≥ 30	5.755	1.195–27.717	0.029	5.151	1.062–24.987	0.042

HR: hazard ratio; CI: confidence interval; CK: creatinine kinase; TIMI: thrombolysis in myocardial infarction; LV: left ventricle; IMR: index of microvascular resistance.

## Data Availability

The data presented in this study are available in Supplementary Material.

## Supplementary Materials

The following supporting information can be downloaded at: https://www.mdpi.com/article/10.3390/jcm13071989/s1.
